# IGFL2‐AS1‐induced suppression of HIF‐1α degradation promotes cell proliferation and invasion in colorectal cancer by upregulating CA9

**DOI:** 10.1002/cam4.5562

**Published:** 2022-12-20

**Authors:** Mengdi Qin, Qiang Liu, Wei Yang, Qiaofeng Wang, Zheng Xiang

**Affiliations:** ^1^ Department of Gastrointestinal Surgery The First Affiliated Hospital of Chongqing Medical University Chongqing China; ^2^ Chongqing Key Laboratory of Department of General Surgery The First Affiliated Hospital of Chongqing Medical University Chongqing China

**Keywords:** CA9, colorectal cancer, degradation, HIF‐1α, IGFL2‐AS1

## Abstract

**Purpose:**

The lncRNA IGFL2‐AS1 is a known cancer‐promoting factor in colorectal cancer (CRC); nonetheless, the mechanism of its carcinogenic effects has not yet been elucidated. This study elaborated on the role and underlying molecular mechanism of IGFL2‐AS1 in promoting CRC cell functions.

**Methods:**

IGLF2‐AS1 expression levels in CRC tissue/normal tissue and CRC cell line/normal colon epithelial cell line were detected by quantitative real‐time polymerase chain reaction. Cell counting kit‐8, colony formation assay, and EdU assay were performed to assess the effect of IGFL2‐AS1 knockdown or overexpression on the proliferative capacity of CRC cells. The migration and invasion abilities of LoVo cells were measured using transwell assay. The expression relationship between IGFL2‐AS1 and carbonic anhydrase 9 (CA9) and the CA9 expression level in CRC tissues and cells was verified by transcriptome sequencing, western blotting, and immunohistochemical staining. Treatment with MG132 and cycloheximide was utilized to explore the mechanism by which IGFL2‐AS1 affects the hypoxia‐inducible factor‐1α (HIF‐1α)/CA9 pathway. A nude mouse xenograft model was constructed to evaluate the effect of IGFL2‐AS1 on CRC growth in vivo.

**Results:**

We discovered that IGFL2‐AS1 was highly upregulated in CRC tumor tissues and cells. IGFL2‐AS1 can functionally promote CRC cell proliferation, migration, and invasion in vitro and accelerate CRC occurrence in vivo. Mechanistic studies demonstrated that IGFL2‐AS1 upregulated the CA9 level by affecting the degradation pathway of HIF‐1α, which elucidates its pro‐proliferative effect in CRC. The lncRNA IGFL2‐AS1 mediated the inhibition of HIF‐1α degradation in CRC and increased CA9 expression, thereby promoting CRC progression.

**Conclusion:**

Our findings suggested that IGFL2‐AS1 is expected to be a promising new diagnostic marker and therapeutic target for CRC.

## Graphical abstract

IGFL2‐AS1 promotes CRC via HIF‐1α/CA9 pathway.

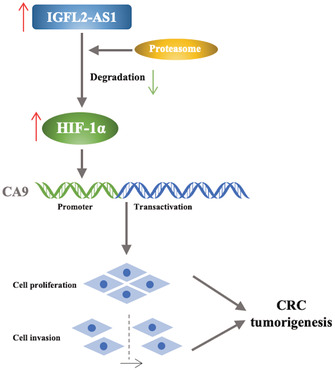

## 1 INTRODUCTION

Colorectal cancer (CRC) is a lethal disease that seriously threatens human health; the incidence and mortality rates of CRC are among the top three of all malignancies worldwide and are increasing annually.^1^ Despite tremendous progress in traditional treatment modalities such as modified surgery and neoadjuvant chemotherapy, the prognosis of CRC remains poor due to its high heterogeneity and recurrence rates.^2^ CRC progression involves changes in colonic mucosal cells, ranging from benign adenomatous polyps to highly heterogeneous advanced invasive adenocarcinomas. The accumulation of genetic mutations and epigenetic modifications are involved in the above‐mentioned multistage tumorigenesis.^3^, ^4^ Hence, clarifying the molecular mechanism underlying CRC initiation and development may provide critical new avenues for improved diagnosis and treatment.

Currently, research on the molecular mechanism of CRC focuses more on protein‐coding genes.^3^ However, the development of high‐throughput sequencing technology has revealed that <2% of genes can encode proteins.^5^ The advent of complete genome sequences has led to the discovery of extensive transcription of noncoding RNAs.^6^ lncRNAs are RNA transcripts over 200 bp in length with little protein‐coding ability. As new players in epigenetics, lncRNAs provide irreplaceable functions in the gene regulation of CRC.^7^ For example, the lncRNA ZFAS1 promotes small nucleolar RNA‐mediated 2’‐O‐methylation through NOP58 recruitment in CRC,^8^ and the lncRNA RAMS11 regulates topoisomerase IIα (TOP2α) to promote metastatic CRC progression.^9^ Although the functions of a few lncRNAs in CRC have been characterized, there is still a great diversity of unknown lncRNAs that may potentially play pivotal roles in CRC.

The lncRNA IGFL2‐AS1 is an antisense RNA of insulin‐like growth factor‐like (IGFL) family member 2, which is located on chromosome 19 and has a transcript of 1665 bp. According to previous studies, IGFL2‐AS1 can promote the Wnt/β‐catenin signaling pathway to activate the progression of tongue squamous cell carcinoma^10^; in breast cancer, IGFL2‐AS1, as a downstream gene of KLF5, promotes IGFL1 expression.^11^ IGFL2‐AS1 can also act as a molecular sponge to competitively bind to miR‐802 and participate in the regulation of gastric cancer.^12^ A microarray analysis of 644 CRC samples from The Cancer Genome Atlas (TCGA) database (https://cancergenome.nih.gov/) showed that IGFL2‐AS1 expression was significantly higher in the stem subtype than in the non‐stem subtype.^13^ A recent study found that IGFL2‐AS1 can promote the proliferation, migration, and invasion of CRC cells in vitro.^14^ However, the molecular mechanism by which IGFL2‐AS1 exerts a tumor‐promoting effect in CRC remains unclear.

Carbonic anhydrase (CA) is a large class of zinc metalloenzymes, and the transmembrane protein CA9 is one of the known tumor‐associated CA isozymes. CA9 is regulated by the transcription factor hypoxia‐inducible factor‐1α (HIF‐1α), which can regulate the pH value inside and outside of tumor cells and indicate the progression of malignant tumors.^15^, ^16^, ^17^, ^18^ Previous studies have confirmed that CA9 can serve as an independent predictor for poor outcomes in CRC.^19^, ^20^

In this study, we aimed to explore the underlying molecular mechanisms by which IGFL2‐AS1 plays a role in promoting CRC progression. Based on functional experiments and the results of transcriptome sequencing analysis, we proposed and validated the hypothesis that IGFL2‐AS1 promotes CRC cell proliferation, migration, and invasion by upregulating CA9.

## 2 MATERIALS AND METHODS

### 2.1 Human tissue samples

This study was conducted in accordance with the principles embodied in the Declaration of Helsinki. The experimental protocol involving human tissues for this study was approved by the Ethics Committee of the First Affiliated Hospital of Chongqing Medical University. Additionally, for this study, written informed consent was obtained from each human tissue provider. Forty pairs of CRC tumor tissue and adjacent non‐tumor tissue samples were collected from patients who had undergone resection of the primary CRC at the First Affiliated Hospital of Chongqing Medical University (Chongqing, China). Samples for this study were collected from 2019 to 2021. Cases were screened according to the following criteria: the resected specimen was confirmed to be CRC by pathological examination, patients did not receive chemotherapy or radiotherapy preoperatively, and patients with hereditary CRC such as Lynch syndrome were excluded. Tissue specimens were immediately frozen in liquid nitrogen after removal and stored at −80°C until use.

### 2.2 Cell lines and culture conditions

Five CRC cell lines, including SW480, SW620, Caco2, HT29, and LoVo, the human normal colon epithelial cell line NCM460, and the 293T cell line were purchased from the Cell Bank of the Chinese Academy of Sciences (Shanghai, China). All cells were cultured in Dulbecco's modified Eagle's medium (DMEM, Gibco, NY, USA) with 10% fetal bovine serum (FBS, Gibco, NY, USA) and 1% penicillin–streptomycin solution (Beyotime, Shanghai, China). The cultural environment was a 37°C incubator with 5% CO_2_. No mycoplasma contamination was found in any of the cell lines, as indicated by mycoplasma detection kit (Lonza, Switzerland).

### 2.3 RNA extraction and real‐time quantitative reverse transcription PCR (qRT‐PCR)

Total RNA from cells and tissues was extracted with Trizol reagent (Invitrogen, CA, USA) and stored at −80°C. RNA concentration and the optical density (OD) value were measured with the Nano‐500 microspectrophotometer (Allsheng, Hangzhou, China). Next, 1 μg RNA was used to synthesize cDNA using the Prime‐Script™ RT kit (Takara, Tokyo, Japan) as directed by the manufacturer's instructions. In addition, 2x SYBR Green qPCR Master Mix (Bimake, TX, USA) was employed for qPCR experiments performed in the ABI StepOne Real‐time Detection System (LTC, Carlsbad, USA). The thermal cycling conditions were as follows: 95°C for 5 min, 90°C for 15 s, 60°C for 45 s, repeating for 40 cycles. There were three replicate holes for each tested sample, and relative RNA levels were compared using the comparative 2^−ΔΔCT^ method. Glyceraldehyde 3‐phosphate dehydrogenase (GAPDH) was amplified as the internal reference. The primer sequences for qRT‐PCR are shown in Table S1 and were synthesized by the TsingKe Company (Chongqing, China).

### 2.4 Subcellular fractionation

The cytoplasmic and nuclear fractions of the HT29 and LoVo cells were isolated using the Minute™ Cytoplasmic and Nuclear Extraction Kit (Invent, Plymouth, USA) in accordance with the instruction manual. Cytoplasmic and nuclear RNA were then extracted with Trizol reagent. qRT‐PCR was performed to measure the relative expression levels of cytoplasmic and nuclear specific RNAs in CRC cells. GAPDH and U6 small nuclear RNA were considered cytoplasmic and nuclear controls, respectively.

### 2.5 Plasmid construction and transfection

Short hairpin RNA (shRNA) directed against human IGFL2‐AS1 and HIF‐1α and negative control short hairpin negative control RNA (sh‐nc) were designed (the sequences are shown in Table S2) and then inserted into the lentiviral vector pGreenPuro (SBI, CA, USA) at the EcoR I/BamH I site. For the overexpression vector (oeIGFL2‐AS1 and oeCA9), the full‐length cDNA of IGFL2‐AS1 and the cDNA sequence containing the CDS region of CA9 was synthesized, PCR‐amplified by the TsingKe Company (Chongqing, China), and ligated into the pCDH‐CMVMCS‐EF1‐CopGFP‐T2A‐puro vector (SBI, CA, USA). It was verified that the nucleotide sequences were correct in all constructed vectors by sequencing.

The packaging plasmid psPAX2, envelope plasmid pMD2.G (Addgene, MA, USA), and 293 T cells were used for lentivirus packaging. The lentiviral vector, auxiliary packaging plasmid, and Lipofectamine 2000 (Invitrogen, CA, USA) were co‐transfected according to the manufacturer's instructions, and the lentiviral supernatant of the 293 T cells was harvested 48 h later. HT29 and LoVo cells were incubated with the viral fluid containing 10 μg/mL polybrene (Solarbio, Beijing, China) for 72 h and subsequently screened with 2 mg/mL puromycin (Beyotime, Shanghai, China) for 14 days to construct stable transfection target plasmid CRC cell lines.

### 2.6 Cell counting kit‐8 (CCK‐8) assay

Cell viability was examined by the cell counting kit (CCK)‐8 assay (Beyotime, Shanghai, China). HT29 and LoVo cells from the control and treatment groups in the logarithmic growth phase were inoculated into 96‐well plates at a density of 2 × 10^3^ cells/well, with three replicate wells for each group. After the cells became adherent (seeding for 6 h), each well was supplemented with 10 μl of CCK‐8 reagent and incubated at 37°C for another 2 h. The OD value of all cells at 450 nm was measured using a spectrophotometric plate reader (BioTek, USA). Next, CCK‐8 reagent was added to the corresponding wells at 24, 48, 72, and 96 h after cell‐plating, and cell viability was further measured according to the OD value.

### 2.7 5‐Ethynyl‐2′‐deoxyuridine (EdU) incorporation assay

Cell proliferation was tested by EdU assay using an EdU Assay Kit (C0075S, Beyotime, Shanghai, China). Following the manufacturer's protocols, 2 × 10^5^ CRC cells that were stably transfected with lentiviral vectors were seeded in 12‐well plates, which were plated with cell climbing slices and cultured overnight. Then, 10 μM EdU working solution was added to the medium and incubated at 37°C for 2 h to complete the EdU labeling. Next, cells in each group were fixed in 4% paraformaldehyde for 15 min. After the fixative was washed off with potassium‐buffered saline (PBS), cells were permeabilized with 0.3% Triton X‐100 for 15 min. After washing, cells were incubated with the Click Reaction Cocktail configured according to the instructions away from light for 30 min. Thereafter, the nuclei were counterstained with Hoechst 33342 for 10 min while avoiding light. Finally, fluorescence images were taken by confocal laser scanning microscopy (Leica, Germany).

### 2.8 Colony formation assay

A total of 2 × 10^3^ cells in the control and treatment groups were seeded in 6‐well plates and cultured in complete medium at 37°C in a humidified atmosphere with 5% CO_2_. The cells were harvested after 14 days and fixed with 4% paraformaldehyde at room temperature for 20 min. After the fixative was washed off with PBS, 0.1% crystal violet was used to stain all cells for 15 min. The cells were washed three times again with PBS buffer, and the number of cell colonies (>50 cells/colony) was counted using ImageJ software (NIH, MD, USA).

### 2.9 Transwell migration and invasion assay

Transwell chambers (Corning, NY, USA) pretreated with Ceturegel (Yeasen, Shanghai, China) at 37°C for 30 min were placed in 24 well plates for detecting the invasion ability of CRC cells. Then, cell suspension containing 5 × 10^4^ LoVo cells that were stably transfected with lentiviral vectors were added to the upper chamber of each well, and 600 μl DMEM containing 10% FBS was added to the bottom well of the chambers. After incubation at 37°C for 24 h, the cells that did not invaded to the bottom of the chambers were wiped off, and 4% paraformaldehyde was used to fix the cells that invaded to the lower face of the chambers. Next, the fixative was washed off with PBS and 0.1% crystal violet was used to stain all cells for 15 min. The migration potential of CRC cells was detected using a similar method but without treating the chambers with Ceturegel. Finally, three random fields in 200× were observed and taken using a bright field microscope (ZEISS, Jena, Germany).

### 2.10 RNA sequencing analysis

Total RNA was extracted from the control group and IGFL2‐AS1 knockdown LoVo cells using Trizol reagent. RNA samples were subjected to RNA Sequencing Analysis by Personalbio Technology Co., Ltd (Shanghai, China).

### 2.11 Protein extraction and western blotting

Total proteins from cells and mice tissues were extracted by ice‐cold RIPA lysis buffer (Beyotime, Shanghai, China) containing 1% phenylmethylsulfonyl fluoride (PMSF, Beyotime, Shanghai, China). The concentration of proteins was determined using the Bicinchoninic Acid Assay (BCA) Kit (Beyotime, Shanghai, China) according to the manufacturer's protocols. Then, the cell lysates were supplied with 5× loading buffer and boiled for 8 min to fully denature the proteins.

Subsequently, 30 μg of the protein sample was separated by 10% sodium dodecyl sulfatepolyacrylamide gel electrophoresis (SDS‐PAGE). After electrophoresis, proteins were transferred onto polyvinylidene difluoride (PVDF) membranes in a semi‐wet system. Membranes were blocked with 5% skimmed milk in 1× Tris Buffered Saline with Tween (TBST) for 120 min at room temperature. After blocking, membranes were incubated with specific primary antibodies against CA9 (1:1000, ab243660, Abcam, Cambridge, UK), HIF‐1α (1:2000, 66,730–1 ‐Ig, Proteintech, Wuhan, China), and GAPDH (1:8000, 10,494‐1‐AP, Proteintech, Wuhan, China) at 4°C overnight (14–16 h). Next, the membranes were washed with 1× TBST three times, followed by incubation with horseradish peroxidase‐labeled goat anti‐rabbit (1:2000, SA00001‐2, Proteintech, Wuhan, China) or goat anti‐mouse secondary antibody (1:2000, Beyotime, Shanghai, China) for 90 min at room temperature, after which any residual antibody was washed away by 1× TBST. Finally, specific protein bands were detected with the enhanced chemiluminescence ECL Kit (Advansta, CA, USA) and visualized with the ChampChemi imaging system (SCS, Beijing, China). All antibodies were diluted in 1× TBST containing 5% bovine serum albumin (BSA). The gray values of all protein bands were analyzed using ImageJ software.

### 2.12 Immunohistochemical (IHC) staining

Human CRC tissues and nude mouse tumor tissues were fixed with 4% paraformaldehyde for 24 h, embedded in paraffin, and cut into 4‐μm‐thick sections. The paraffin sections were baked at 60°C for 2 h and dewaxed in fresh xylene for 30 min. Then, the slides were hydrated with gradient alcohol, followed by boiling them in a pressure cooker containing sodium citrate for 2 min to retrieve the antigens. Next, sections cooled to room temperature were incubated with an endogenous peroxidase blocking agent for 15 min and blocked with goat serum blocking solution for 30 min. The slices were incubated with specific primary antibodies against CA9 (1:2000, ab243660, Abcam, Cambridge, UK), HIF‐1α (1:300, 66,730‐1‐Ig, Proteintech, Wuhan, China), and Ki67 (1:500, ab92742, Abcam, Cambridge, UK) overnight (14–16 h) at 4°C. After washing with PBS buffer, the slices were incubated with biotin‐labeled secondary antibodies for 30 min and subsequently incubated with horseradish enzyme‐labeled chain avidin solution for 30 min. Finally, positive staining was visualized with brown 3,3′‐diaminobenzidine tetrahydrochloride (DAB, ZSGB, Beijing, China), and counterstaining was performed with hematoxylin. The sections were mounted with 60% neutral resin. The stained sections were scanned with the Pannoramic DESK scanner (3DHISTECH, Budapest, Hungary). The images in 200× and 400× were observed and acquired with the CaseViewer 2.4 software module (3DHISTECH, Budapest, Hungary).

### 2.13 Xenograft mouse model

The animal experiment protocol of this study was authorized by the Animal Experiment Ethics Committee of Chongqing Medical University. Male BALB/c‐nu nude mice aged 4–5 weeks were purchased from HFK Bioscience Co., Ltd. (Beijing, China). All animals were divided randomly into four groups (*n* = 3 mice per group) and reared under SPF‐grade sterile conditions in the Laboratory Animal Center of Chongqing Medical University. Next, 1 × 10^7^ LoVo cells stably transfected with sh‐nc, shIGFL2‐AS1#1, pCDH, and oe IGFL2‐AS1 were resuspended in 100 μl of PBS solution and subcutaneously injected into the right armpit of the nude mice in each group. Tumor volumes were measured every 4 days from day 7, which was calculated as follows: volume (mm^3^) = 0.5 × (largest diameter) × (smallest diameter)^2^. On day 27, all mice were sacrificed by cervical dislocation; the tumor tissues were collected, weighed, and photographed. Proteins from tumor tissues were extracted as previously described, and IHC staining was conducted on mice tumor sections.

### 2.14 Statistical analysis

Statistical analysis was performed with GraphPad Prism 8 software (GraphPad Software, CA, USA). Pearson's chi‐square test was used to examine the association between IGFL2‐AS1 expression and clinicopathological characteristics of CRC patients. The RNA expression level in paired CRC tumor tissues was analyzed using the paired Student's t‐tests. The differences between two independent groups were analyzed using the unpaired two‐tailed Student's t‐test. The comparisons between multiple groups were performed using ANOVA, and the Tukey test was used for post hoc comparisons. All results were presented as means± standard deviation (SD). The differences were considered significant for *p*‐values <0.05. Significance is indicated as follows: *p < 0.05, **p < 0.01 and ***p < 0.001.

## 3 RESULTS

### 3.1 IGFL2‐AS1 was significantly upregulated in CRC and predominantly localized in the cytoplasm

**FIGURE 1:**
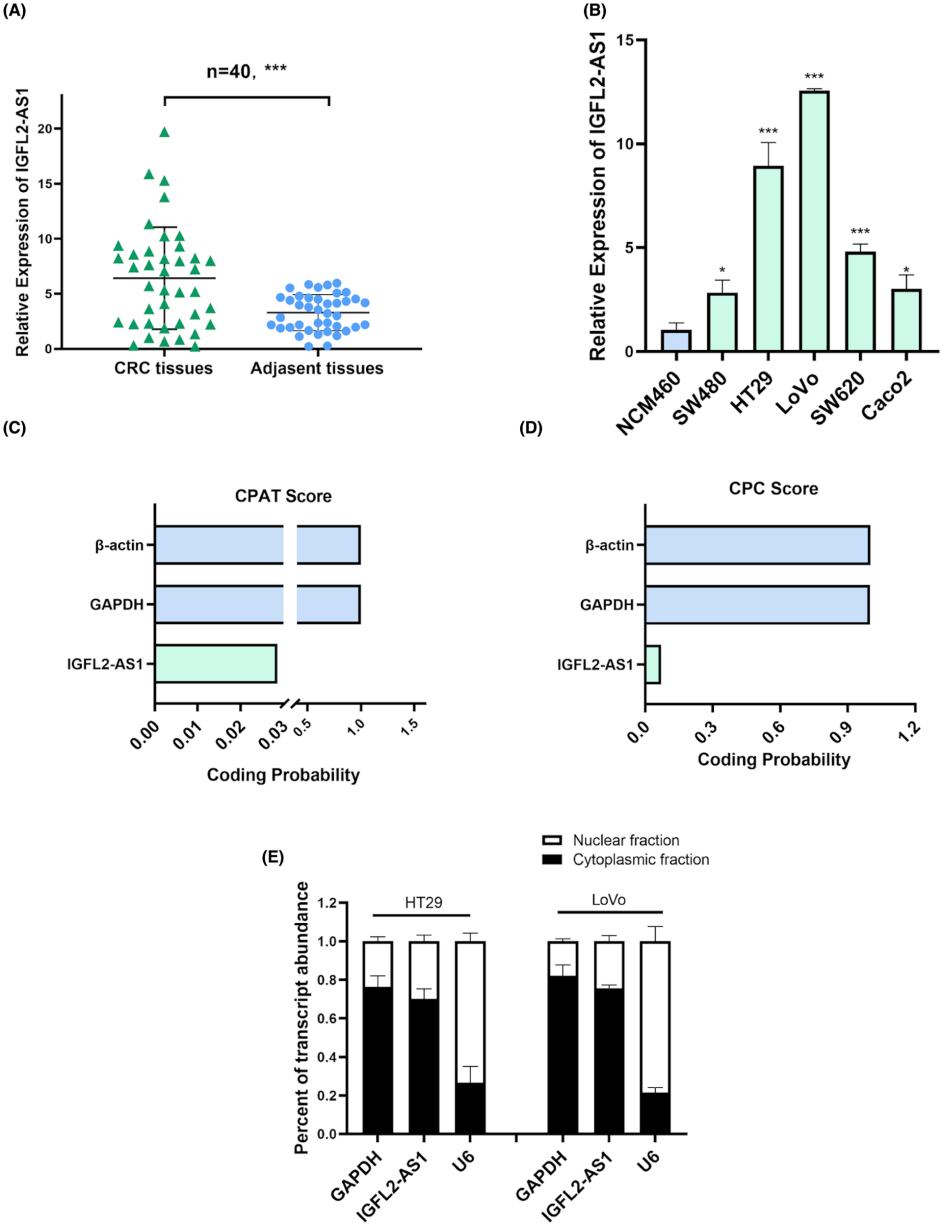
IGFL2‐AS1 expression level in CRC. (A) IGFL2‐AS1 expression in 40 pairs of colorectal cancer tumor tissues and adjacent normal tissues. (B) IGFL2‐AS1 expression in five colorectal cancer cell lines and normal colon epithelial cell lines. GAPDH was used as the internal control. (C, D) The protein‐coding ability of IGFL2‐AS1 was detected using CPAT (C) and CPC (D). (E) The subcellular localization of IGFL2‐AS1 in HT29 and LoVo cells. (*p < 0.05, **p < 0.01, ***p < 0.001)

To identify IGFL2‐AS1 expression in CRC, qRT‐PCR was performed to evaluate the IGFL2‐AS1 levels in 40 pairs of CRC tumors and adjacent non‐cancer tissues. The results revealed that IGFL2‐AS1 expression was significantly higher in tumor tissues than in adjacent non‐tumor tissues (Figure 1A). In addition, based on our data analysis of 479 CRC tissues and 42 normal colon tissues downloaded from the TCGA database on April 14, 2021, we found that IGFL2‐AS1 was prominently upregulated in tumor tissues (Table S3). We subsequently quantified IGFL2‐AS1 expression levels in the normal colon epithelial cell line NCM460 and a panel of CRC cell lines, including SW480, SW620, HT29, LoVo, and Caco2. According to the results, it appeared to be more highly expressed in CRC cell lines than in the NCM460 cell line (Figure 1B). We selected HT29 and LoVo cells, which expressed relatively high IGFL2‐AS1 levels, for further experiments.

Further, we analyzed the association between the clinicopathological characteristics and the RNA level of IGFL2‐AS1 in these 40 CRC patients. According to the results of qRT‐PCR, we found the median value of IGFL2‐AS1 expression in 40 pairs of tissues and then divided all patients into two groups: IGFL2‐AS1 high expression group and IGFL2‐AS1 low expression group. We found that the expression level of IGFL2‐AS1 was considerably associated with tumor size and TNM stage, but there was no significant correlation with the patient sex, age, and tumor location (Table 1).

**TABLE 1:** Correlation between IGFL2‐AS1 expression and clinicopathologic characteristics in 40 CRC patients.

Parameters	Total case	IGFL2‐AS1		*p*‐value
		High expression	Low expression	
	40	20	20	
Gender				0.7515
Male	21	10	11	
Female	19	10	9	
Age(Years)				0.3112
>50	27	15	12	
≤50	13	5	8	
Location				0.1134
Colon	21	8	13	
Rectum	19	12	7	
Stages				0.0467*
I‐II	14	4	10	
III‐IV	26	16	10	
Metastasis				0.3373
Yes	23	13	10	
No	17	7	10	
Tumor size				0.0098**
>40 mm	16	12	4	
≤40 mm	24	8	16	

* *p* < 0.05

** *p* < 0.01.

Next, online protein‐coding potential assessment software, the Coding Potential Assessment Tool (CPAT, http://lilab.research.bcm.edu/cpat/) and the Coding Potential Calculator (CPC, http://cpc.cbi.pku.edu.cn/) were used to estimate the protein‐coding potential of IGFL2‐AS1, and it was found that it is unlikely for IGFL2‐AS1 to encode any protein (Figure 1C, D). Furthermore, the results of subcellular fractionation confirmed that IGFL2‐AS1 was primarily located in the cytoplasm of HT29 and LoVo cells (Figure 1E). Taken together, these data indicate that IGFL2‐AS1 was generally highly expressed in CRC and had the potential to be a molecular target of CRC.

### 3.2 IGFL2‐AS1 knockdown decreased CRC cell proliferation, migration, and invasion in vitro*,* whereas IGFL2‐AS1 overexpression showed an enhanced effect

**FIGURE 2:**
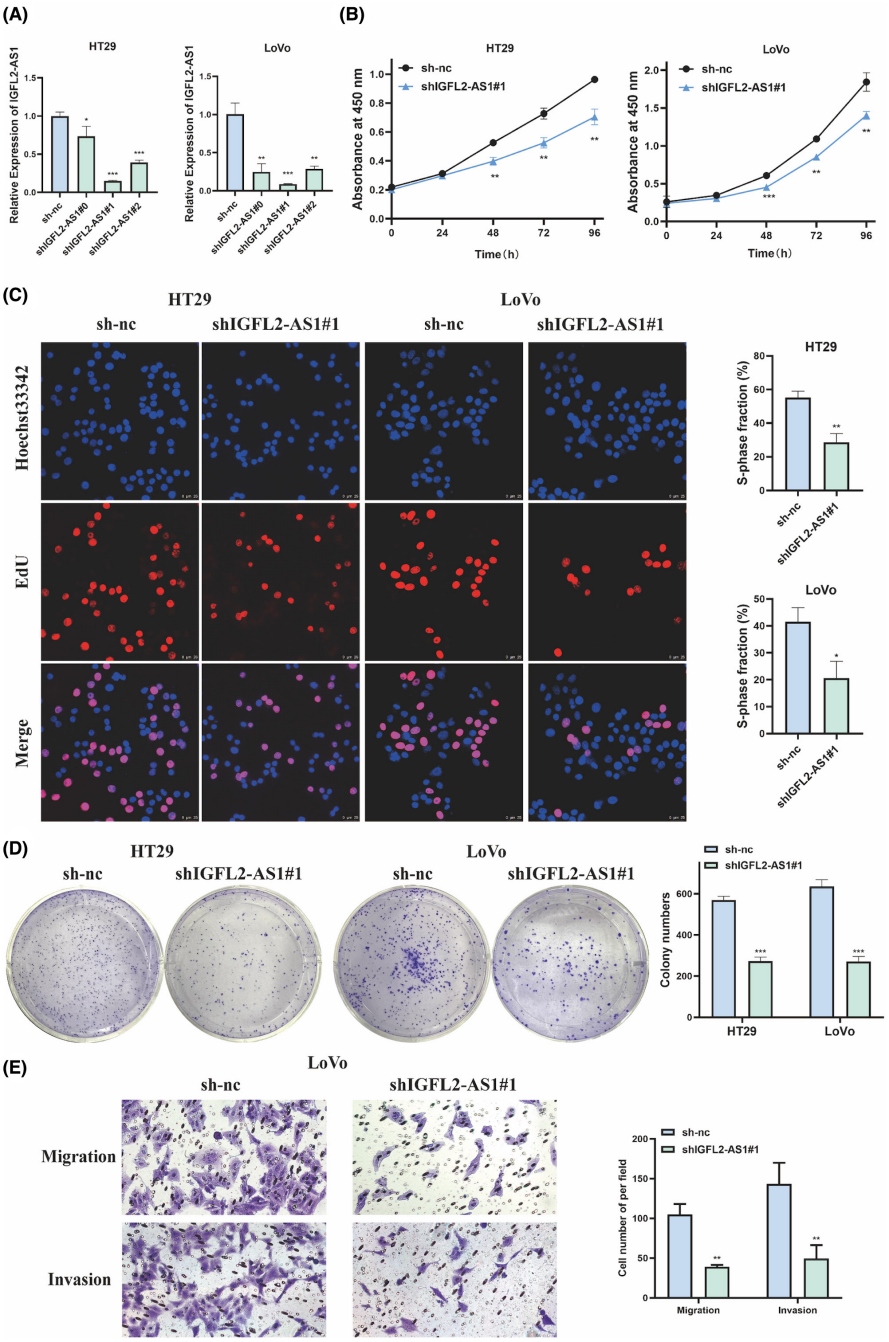
IGFL2‐AS1 knockdown inhibits CRC cell growth, migration, and invasion in vitro. (A) The knockdown efficiency of IGFL2‐AS1 in HT29 and LoVo cells was verified by qRT‐PCR. (B) CCK‐8 assay was utilized to detect the effect of IGFL2‐AS1 knockdown on the viability of HT29 and LoVo cells. (C, D) The proliferation ability of HT29 and LoVo cells in the IGFL2‐AS1 knockdown group and negative control group was analyzed by EdU assay (C) and clone formation assay (D). (E) Transwell assay was performed to measure the effect of IGFL2‐AS1 downregulation on LoVo cell migration and invasion. (magnification, 200× and 400×; scale bar, 25 μm; *p < 0.05, **p < 0.01, ***p < 0.001)

Given the considerably differential IGFL2‐AS1 expression in CRC, we speculated that it may play an active biological role in CRC. Therefore, we constructed CRC cell lines with stable IGFL2‐AS1 knockdown. Three shRNA sequences were designed against IGFL2‐AS1, and HT29 and LoVo cells were transfected with lentiviral vectors carrying shRNA and empty control sh‐nc, respectively. qRT‐PCR was utilized to evaluate the knockdown efficiency of these three shRNAs. It can be seen that shIGFL2‐AS1#1 knockdown affected IGFL2‐AS1 expression to the greatest extent (Figure 2A). The cell viability was examined by the CCK‐8 assay. The results showed that the viability of HT29 and LoVo cells was markedly reduced in the shIGFL2‐AS1#1 group compared to the sh‐nc group (Figure 2B). To further clarify the effect of IGFL2‐AS1 on CRC cell proliferation, we performed EdU and colony formation experiments. The results show that the deletion of IGFL2‐AS1 significantly reduced the proliferation rate and colony formation capacity of HT29 and LoVo cells (Figure 2C, D). Further, the result of transwell assay revealed that IGFL2‐AS1 knockdown highly reduces the migratory and invasive ability of LoVo cells (Figure 2E).

**FIGURE 3:**
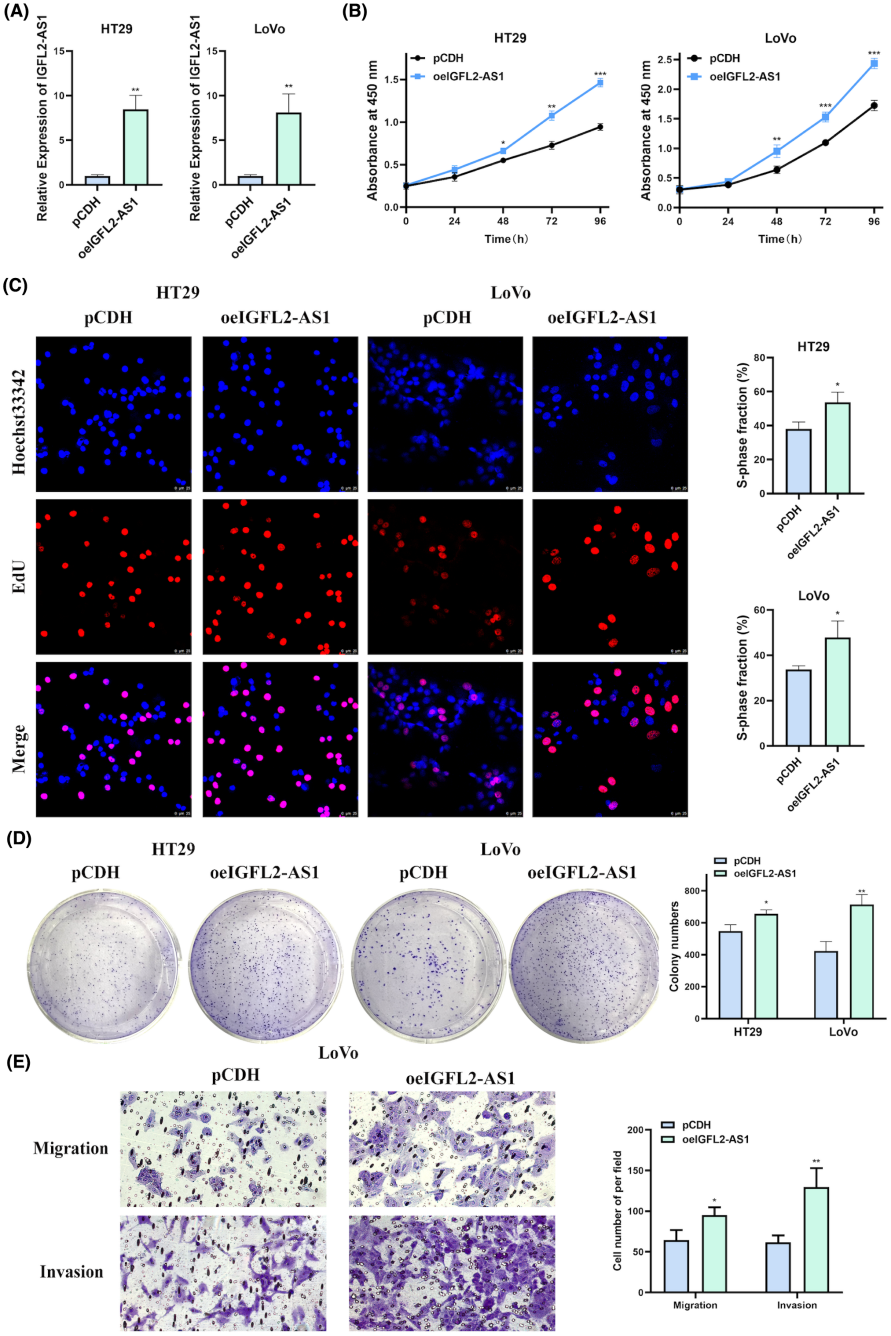
IGFL2‐AS1 overexpression promotes CRC cell proliferation, migration, and invasion in vitro. (A) qRT‐PCR was employed to assess the overexpression efficiency in CRC cells. (B) The difference in cell viability of CRC cells in the IGFL2‐AS1 overexpressing group and control group was measured by CCK‐8 assay. (C, D) The effect of IGFL2‐AS1 overexpression on CRC cell proliferation was detected by EdU assay (C) and clone formation assay (D). (E) The migratory and invasive abilities of LoVo cells in the IGFL2‐AS1 overexpressing group and control group were analyzed using transwell assay. (magnification, 200× and 400×; scale bar, 25 μm; *p < 0.05, **p < 0.01, ***p < 0.001)

In addition, we transfected HT29 and LoVo cells with the overexpression vector of IGFL2‐AS1 and control vector pCDH. qRT‐PCR results confirmed the successful IGFL2‐AS1 overexpression (Figure 3A). Thereafter, CCK‐8, EdU, and colony formation assays were used to detect the viability and proliferation of HT29 and LoVo cells. In contrast, after exogenous IGFL2‐AS1 overexpression, the viability of CRC cells was considerably increased (Figure 3B) and proliferation was enhanced (Figure 3C, D) compared with those of control cells. Furthermore, transwell assay illustrated that the overexpression of IGFL2‐AS1 increased the migratory and invasive potential of LoVo cells (Figure 3E). But no significant changes in apoptosis ability have been ovserved in LoVo cells when IGFL2‐AS1 knockdown or overexpression compared to corresponding control groups (Figure S1). Collectively, these results suggest that IGFL2‐AS1 can promote CRC cell proliferation, migration, and invasion in vitro.

### 3.3 IGFL2‐AS1 positively regulated CA9 expression in CRC

**FIGURE 4:**
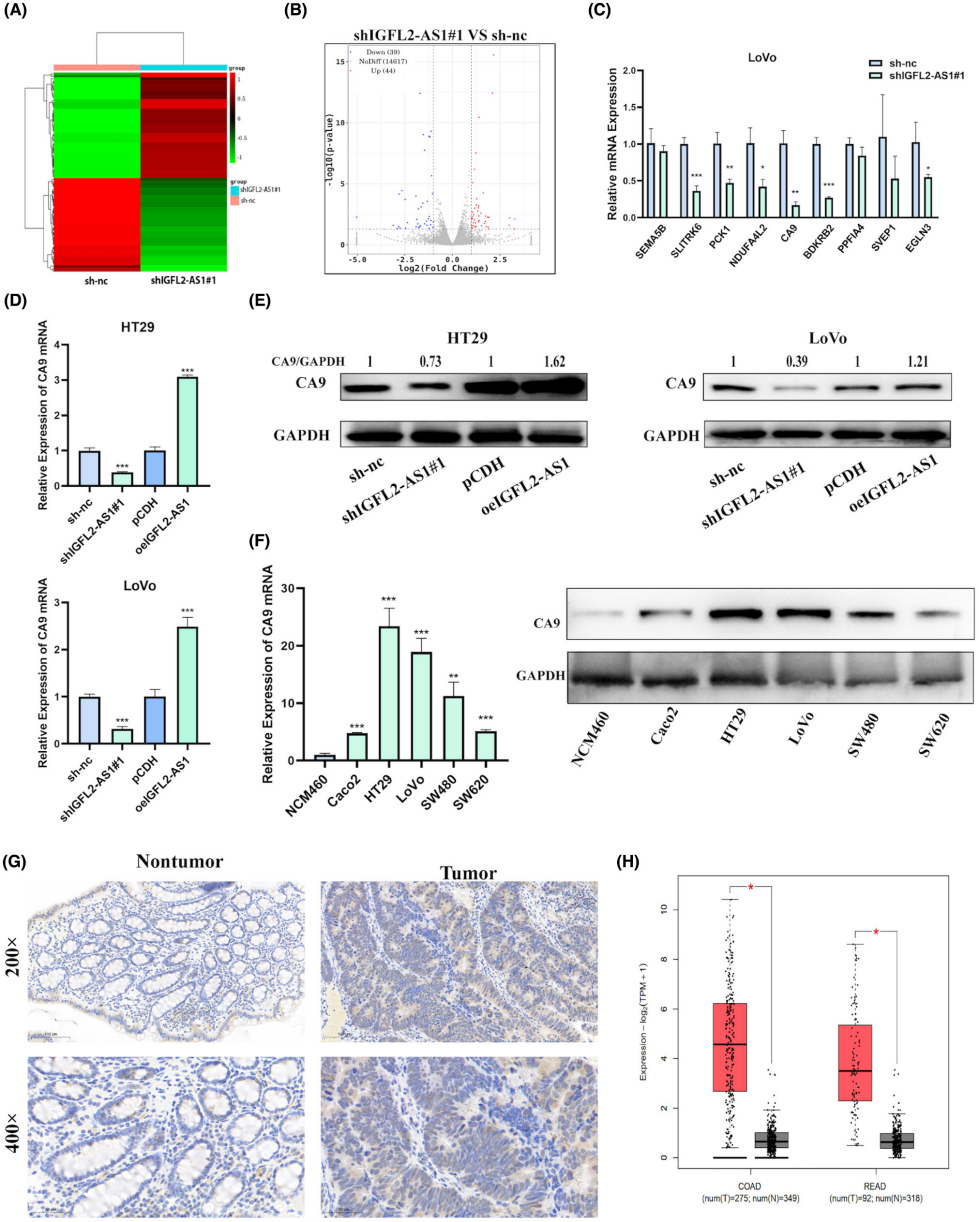
IGFL2‐AS1 positively regulates CA9 expression in CRC. (A, B) Differential gene heat map (A) and volcano map (B) of negative control and IGFL2‐AS1 knockdown LoVo cell populations. Upregulation is shown as red, and downregulation is shown as green. (C) mRNA levels of 9 downregulated genes in IGFL2‐AS1 knockdown LoVo cells were measured by qRT‐PCR. (D, E) qRT‐PCR and western blotting were utilized to detect mRNA (D) and protein (E) levels of CA9 in HT29 and LoVo cells that overexpress or knockdown IGFL2‐AS1. (F) The expression level of CA9 in normal colonic epithelial cell line and CRC cell lines were detected by qPCR and western blotting. (G) Immunohistochemistry was performed to assess the protein level of CA9 in CRC tumor tissue and adjacent non‐tumor tissue. (H) CA9 expression in TCGA‐derived specimen datasets in the GEPIA database. (magnification, 200× and 400×; scale bar, 100 and 50 μm; *p < 0.05, **p < 0.01, ***p < 0.001)

The previous experiments confirmed that IGFL2‐AS1 was principally localized in the cytoplasm (Figure 1E), suggesting that IGFL2‐AS1 may have a role in the cytoplasm. To probe the molecular mechanism by which IGFL2‐AS1 promotes CRC cell function, we identified the gene expression profiles of IGFL2‐AS1 knockdown and control group LoVo cells by RNA sequencing analysis. Hierarchical clustering analysis results revealed that the mRNA levels between two of these groups were distinguishable (Figure 4A). As shown in the volcano plot (Figure 4B), the IGFL2‐AS1 knockdown cell population had 39 downregulated mRNAs and 44 upregulated mRNAs compared to the control cells (|log2 fold change| > 1, *p*‐value <0.05). We then examined the expression levels of nine downregulated mRNAs that may be associated with malignant progression and poor prognosis in CRC by qRT‐PCR in LoVo cells. We found that *CA9* was the most drastically reduced gene in IGFL2‐AS1 knockdown LoVo cells (Figure 4C). In view of this phenomenon, we hypothesized that IGFL2‐AS1 may have an expression and functional relationship with CA9.

To test this hypothesis, qRT‐PCR and western blotting were used to assess the effect of IGFL2‐AS1 on CA9 expression in HT29 and LoVo cells. The results illustrated that IGFL2‐AS1 knockdown considerably reduced the mRNA and protein levels of CA9, which were significantly increased after IGFL2‐AS1 overexpression (Figure 4D, E). Subsequently, we found that the mRNA and protein levels of CA9 in five CRC cell lines were markedly increased than those in the normal colon epithelial cell line NCM460 (Figure 4F). In addition, IHC staining was performed to evaluate the protein expression of CA9 in CRC tissues and non‐tumor tissues, and the results showed that CA9 was upregulated in tumor tissues (Figure 4G). Similarly, from a sample analysis of the GEPIA online database (http://gepia2.cancer‐pku.cn), we observed that CA9 expression was remarkably higher in CRC tissues than in normal tissues (Figure 4H). The above results indicate that CA9 is highly expressed in CRC and positively regulated by IGFL2‐AS1.

### 3.4 CA9 was required for IGFL2‐AS1 to enhance CRC cell growth, migration, and invasion in vitro

**FIGURE 5:**
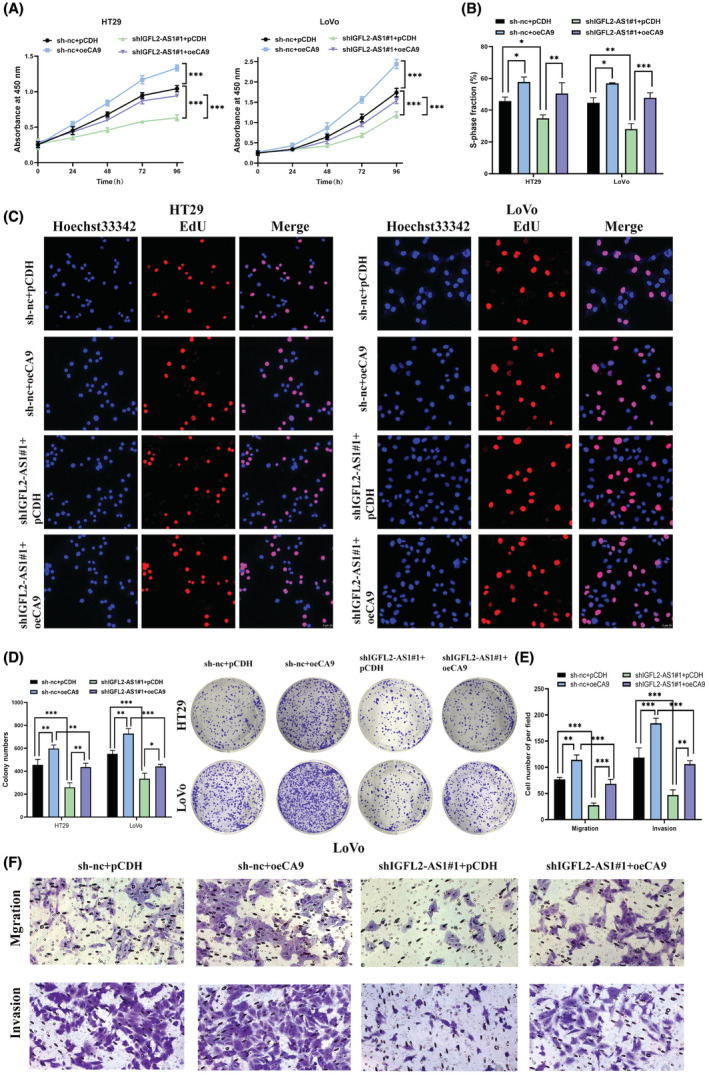
CA9 is required for IGFL2‐AS1 to enhance CRC cell growth, migration, and invasion in vitro. (A) The effect of CA9 regulated by IGFL2‐AS1 on CRC cell viability was detected by CCK‐8 assay. (B, C) The quantitative results and images of the EdU assay showing that IGFL2‐AS1 regulates CRC cell proliferation through CA9. (D) The effect of CA9 overexpression and IGFL2‐AS1 knockdown co‐transfection on CRC cell proliferation was examined using a colony formation assay. (E, F) The effect of migration and invasion of IGFL2‐AS1 and CA9 co‐transfection in LoVo cells was determined by transwell assay. (magnification, 200× and 400×; scale bar, 25 μm; *p < 0.05, **p < 0.01, ***p < 0.001)

As previously described, CA9 is regulated by IGFL2‐AS1 and is an important prognostic marker for CRC.^19^, ^20^ We postulate that the regulatory role of IGFL2‐AS1 on CA9 expression is the reason for its promotive effect on CRC cell proliferation, migration, and invasion. To this end, we co‐transfected the overexpressing CA9 vector, oeCA9, and the IGFL2‐AS1 knockdown vector. Transfection efficiency was verified by qRT‐PCR (Figure S2). As speculated, the CCK‐8 assay demonstrated that the CA9 overexpression enhanced the cell viability of HT29 and LoVo cells compared to controls (Figure 5A). Similarly, EdU and colony formation assays were employed to confirm that the CA9 overexpression promoted cell proliferation (Figure 5B–D). In addition, transwell assay demonstrated that CA9 overexpression enhanced the migration and invasion of LoVo cells (Figure 5E, F).

Interestingly, although the absence of IGFL2‐AS1 clearly impairs HT29 and LoVo cell viability when the overexpressing CA9 vector was co‐transfected with shIGFL2‐AS1#1, the inhibition of CRC cell viability by IGFL2‐AS1 knockdown was blocked as CA9 level increased (Figure 5A). Similarly, EdU and colony formation assay showed that IGFL2‐AS1 knockdown reversed the promotive effect of CA9 overexpression on CRC cell proliferation (Figure 5B–D). Subsequently, transwell assay was performed to confirm that IGFL2‐AS1 knockdown can block the facilitation of the migration and invasion of LoVo cells by CA9 overexpression (Figure 5E, F). In short, these data indicate that CA9 is an integral factor for IGFL2‐AS1 to play a carcinogenic role in CRC.

### 3.5 IGFL2‐AS1 upregulated CA9 expression by inhibiting HIF‐1α proteolysis

**FIGURE 6:**
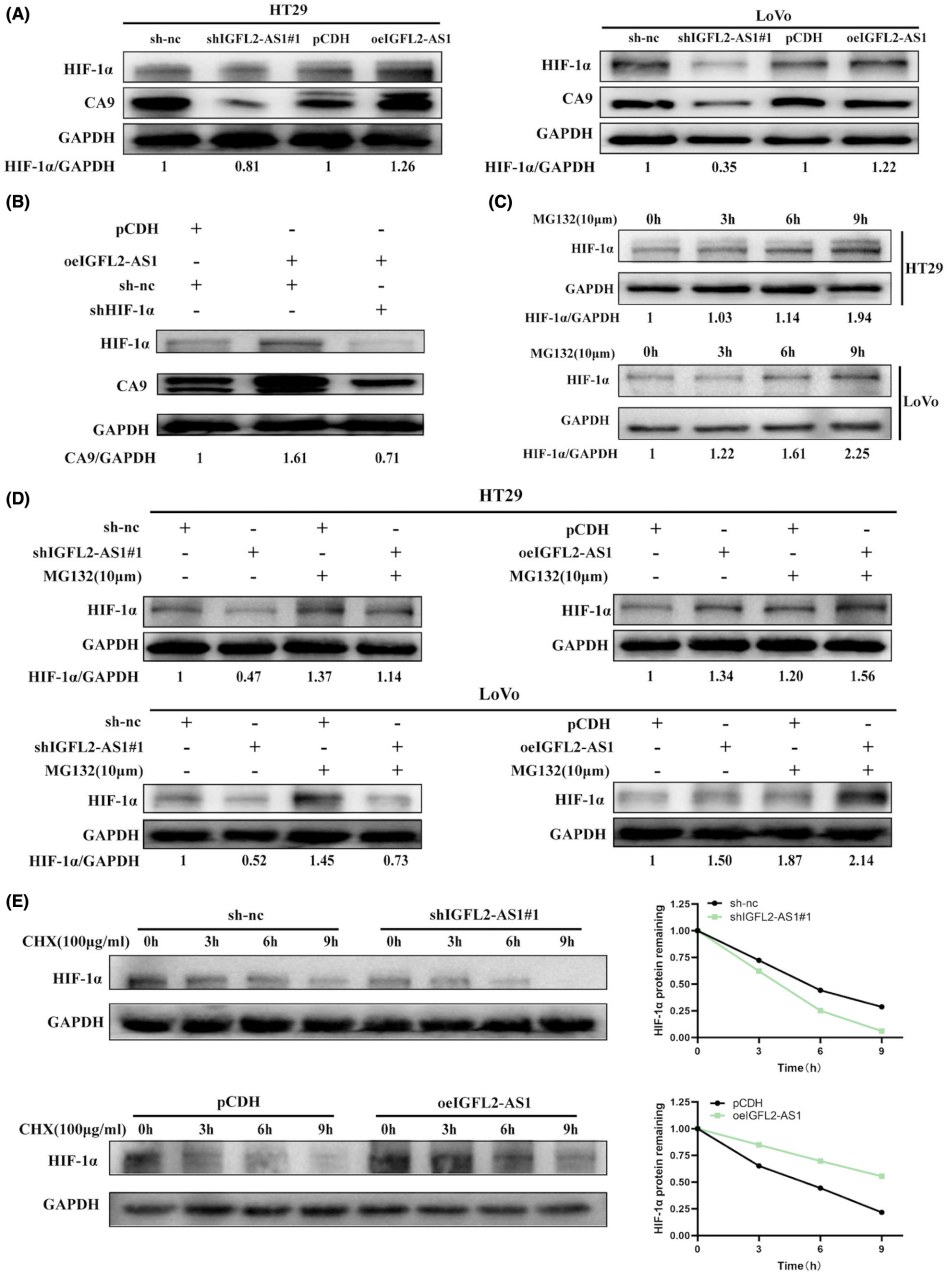
IGFL2‐AS1 upregulates CA9 expression by inhibiting HIF‐1α proteolysis. (A) Western blotting was used to assess the HIF‐1α and CA9 expression levels in CRC cells with IGFL2‐AS1 knockdown and overexpression. (B) The effect of HIF‐1α knockdown on the increase in CA9 protein level by IGFL2‐AS1 overexpression. (C) The protein levels of HIF‐1α after HT29 and LoVo cells were treated with MG132 (10 μM) for 0, 3, 6, and 9 h and were measured by western blotting. (D) CRC cells were transfected with sh IGFL2‐AS1#1, oe IGFL2‐AS1, or negative control, and treated with or without MG132 (10 μM) for 6 h. The protein levels of HIF‐1α were detected by western blotting. (E) CRC cells in IGFL2‐AS1 knockdown and overexpression groups were treated with CHX (100 μg/mL) for 0, 3, 6, and 9 h, and western blotting was performed to measure the HIF‐1α expression levels

Given that previous studies have confirmed that HIF‐1α can bind to the upstream promoter immediately adjacent to the CA9 transcription initiation site, it may be the main transcriptional regulator of CA9.^18^, ^21^ To explore the molecular mechanism underlying the regulation of CA9 by IGFL2‐AS1, we speculated that IGFL2‐AS1 might affect HIF‐1α expression in CRC cells. Western blotting was performed to test this hypothesis. The results showed that in HT29 and LoVo cells, the HIF‐1α protein level was decreased in the IGFL2‐AS1 knockdown groups and increased in the IGFL2‐AS1 overexpression groups (Figure 6A). Furthermore, the promotion of CA9 expression by IGFL2‐AS1 overexpression was suppressed after HIF‐1α knockdown in LoVo cells (Figure 6B). These data show that HIF‐1α may be involved in mediating the regulation of CA9 expression by IGFL2‐AS1.

To further clarify howthe manner in which IGFL2‐AS1 affects HIF‐1α, we examined the mRNA expression of HIF‐1α in knockdown and overexpressing IGFL2‐AS1 cells using qRT‐PCR. The results indicated no significant changes in the mRNA level of HIF‐1α (Figure S3), which was consistent with the result of RNA sequencing analysis, suggesting that IGFL2‐AS1 may primarily affect the post‐transcriptional regulation of HIF‐1α. Under normoxic conditions, prolyl‐hydroxylated HIF‐1α can bind to the tumor suppressor protein von Hippel–Lindau (VHL) and is recognized by E3 ubiquitinated protein ligase for proteasomal degradation.^22^, ^23^ Therefore, to validate the effect of the proteasome system on the HIF‐1α expression level in CRC cells, we treated HT29 and LoVo cells separately for a period of time with specific concentrations of MG132 (Selleck, USA), a proteasome inhibitor. Results of western blotting indicated that HIF‐1α protein content accumulated over time (Figure 6C), suggesting that MG132 could effectively prevent the degradation of HIF‐1α by the ubiquitin‐proteasome system in CRC cells. Subsequently, CRC cells transfected with sh‐nc, shIGFL2‐AS1#1, pCDH, and oe IGFL2‐AS1 were incubated with the same concentration of MG132 for 6 h, and western blotting was used to measure the change in HIF‐1α protein level. Interestingly, we found that cells in the MG132‐treated groups had significantly higher HIF‐1α levels than those in the non‐MG132‐treated groups. Furthermore, MG132‐induced HIF‐1α accumulation was more pronounced in cells overexpressing IGFL2‐AS1, while MG132 induced less HIF‐1α accumulation in IGFL2‐AS1 knockdown cells compared to the respective control groups (Figure 6D). This suggests that IGFL2‐AS1 can positively regulate HIF‐1α expression by inhibiting the degradation by the proteasome system. In addition, the protein synthesis inhibitor cycloheximide (CHX, MCE, Shanghai, China) was employed to treat LoVo cells with stable IGFL2‐AS1 knockdown or overexpression for a period of time. Results of western blotting indicated that IGFL2‐AS1 knockdown accelerated the degradation of HIF‐1α, whereas IGFL2‐AS1 overexpression slowed down the degradation of HIF‐1α protein (Figure 6E). Collectively, these results demonstrate that IGFL2‐AS1 may affect the proteolysis pathway of HIF‐1α and may increase the expression of its downstream gene *CA9* by inhibiting the degradation of HIF‐1α.

### 3.6 IGFL2‐AS1 accelerated CRC tumor growth in vivo via the HIF‐1α/CA9 pathway

**FIGURE 7:**
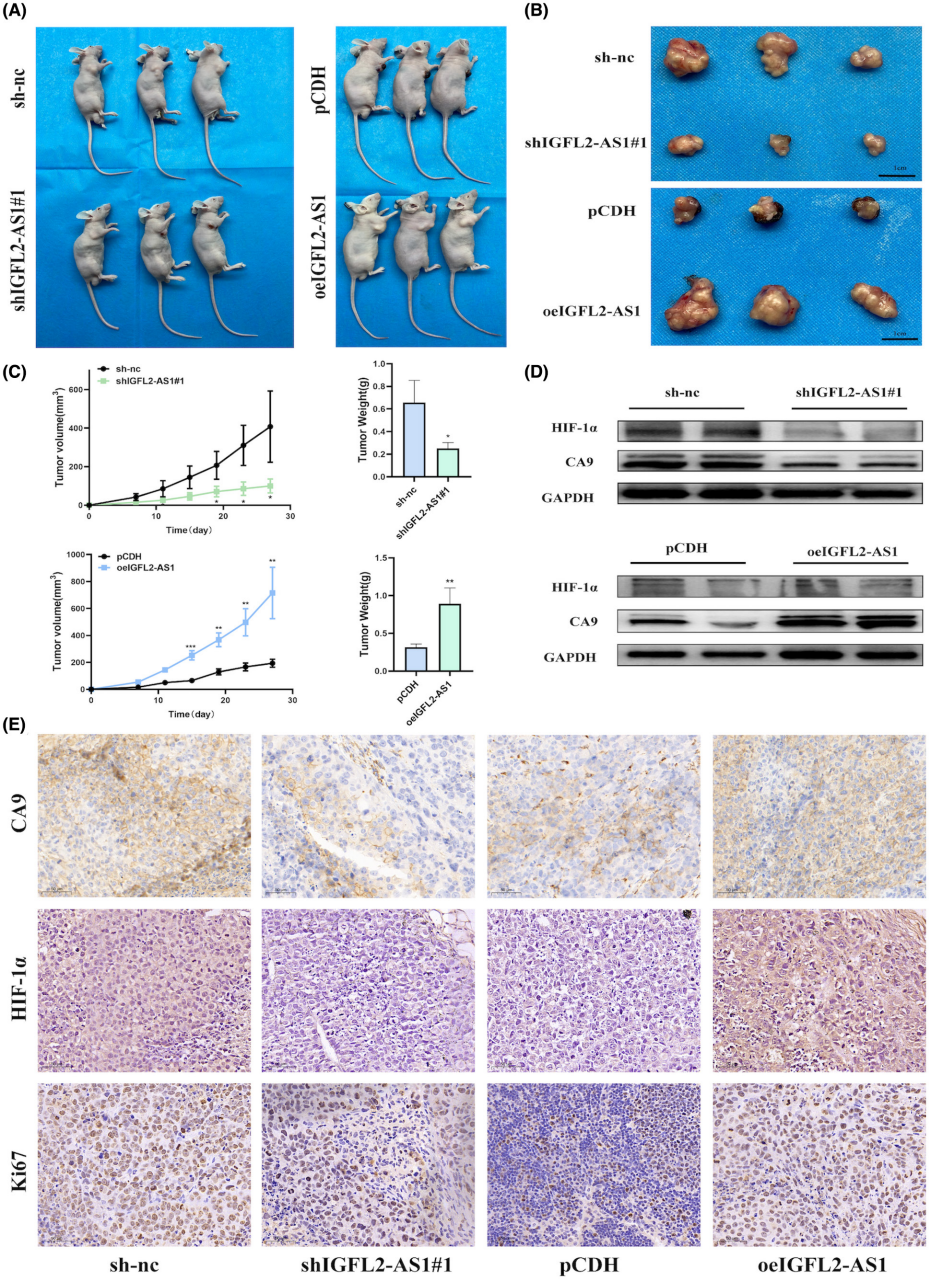
IGFL2‐AS1 accelerates CRC tumor growth in vivo. (A, B) All mice were sacrificed on day 27 after injection, and tumors were harvested and photographed. (C) The histogram of tumor weight and the tumor growth curve is based on measured tumor volume. (D) Western blotting was performed to verify the protein levels of HIF‐1α and CA9 in the tumors of nude mice in each group. Two samples were taken from each group. (E) Immunohistochemical staining of HIF‐1α, CA9, and Ki67 in xenograft tumors. (scale bar, 1 cm; *p < 0.05, **p < 0.01, ***p < 0.001)

As discussed above, we demonstrated that IGFL2‐AS1 can promote CRC cell growth in vitro by regulating CA9 expression. Further, a xenograft tumor model of nude mice was established to verify whether IGFL2‐AS1 could accelerate tumor progression in vivo. LoVo cells with stable knockdown or overexpression of IGFL2‐AS1 and control cells were injected subcutaneously into the right axilla of nude mice (three nude mice in each group). From day 7, the tumor size was measured every 4 days until the nude mice were sacrificed by cervical dislocation, and the tumors were harvested on day 27 (Figure 7A, B). As expected, slower tumor growth, smaller tumor volume, and lighter tumor weight were observed in mice in the shIGFL2‐AS1#1 group than those in the sh‐nc group. Similarly, compared to the control group, the tumor volume, weight, and growth rate of mice in the oe IGFL2‐AS1 group were considerably higher (Figure 7C). This evidence illustrates that IGFL2‐AS1 can indeed promote CRC cell proliferation in vivo.

In addition, western blotting and IHC staining were utilized to detect the protein levels of HIF‐1α and CA9 in tumor tissues from the nude mice in each group. The results of western blotting and IHC were consistent, in which IGFL2‐AS1 knockdown in nude mouse tumor tissues reduced the HIF‐1α and CA9 expression, whereas IGFL2‐AS1 overexpression had the opposite results (Figure 7D, E). Unsurprisingly, IHC staining indicated that the proliferation marker Ki67 decreased with IGFL2‐AS1 knockdown but increased in the tumor tissues of nude mice overexpressing IGFL2‐AS1 (Figure 7E). Taken together, these results confirm that IGFL2‐AS1 can maintain CRC tumor growth through the HIF‐1α/CA9 pathway in vivo.

## 4 DISCUSSION

The high relapse and metastasis rates of CRC lead to poor outcomes and low long‐term survival.^24^ In the face of limited treatment options for advanced CRC, it is crucial to clarify the molecular mechanisms behind CRC occurrence and development.^3^, ^4^ The next‐generation sequencing technology has made it possible to identify the specific expression patterns of noncoding RNAs in various tumors, including CRC, and the possibility that lncRNAs could become novel biomarkers and therapeutic targets for CRC.^25^, ^26^

Based on the microarray analysis of CRC clinicopathological samples downloaded from the TCGA database, we found that the lncRNA IGFL2‐AS1 was drastically upregulated in CRC tissues (Table S4). Likewise, qRT‐PCR confirmed that IGFL2‐AS1 showed a significant overexpression trend in CRC tissues and cell lines compared to paired adjacent non‐tumor tissues and normal colon epithelial cell lines. This gives us reason to speculate that IGFL2‐AS1 may play an active biological role in the pathophysiology of CRC. Unsurprisingly, IGFL2‐AS1 knockdown considerably reduced the viability of CRC cells, turning them into less proliferative cells both in vivo and in vitro. Conversely, CRC cells stably overexpressing IGFL2‐AS1 had a greater proliferative, migratory, and invasive capacity than controls. These conclusions are consistent with the phenomenon observed in the previous study,^14^ and further complement the results of experiments in vivo, providing more valuable evidence for the idea that *IGFL2‐AS1* may be an oncogene in CRC.

Based on the mechanistic basis provided by the functional experiments, transcriptome sequencing analysis was utilized to explore the downstream genes of IGFL2‐AS1. We validated the gene with the most sharply reduced mRNA level, *CA9*, in the IGFL2‐AS1 knockdown cell population. In a variety of solid tumors, including CRC,^19^, ^20^ bladder cancer,^27^ non‐small cell lung cancer,^28^ triple‐negative breast cancer,^29^ and pancreatic cancer,^30^ CA9 has been widely confirmed as an oncogenic factor and diagnostic molecular marker. In the present study, we demonstrated that IGFL2‐AS1 could positively regulate CA9 expression in vitro and in vivo and promote CRC development by upregulating CA9. CA9, which has a catalytic domain facing outside the cell membrane, is an enzyme that plays a central role in tumor pH regulation, and it catalyzes the balance between H^+^, CO_2_, and HCO_3_^−^ inside and outside the cell.^31^ Since the accumulation of lactate resulting from increased glycolytic activity in the tumor microenvironment often activates the transmembrane transporters such as CA9,^32^ the CA9 expression level may be considered a marker of tumor malignancy. In addition, CA9 can also promote the transport activity of the monocarboxylic transporter (MCT) through non‐catalytic interactions, which can mediate the release of large amounts of lactate and protons from highly glycolytic tumor cells.^15^, ^33^ The regulatory effect of CA9 on the acid–base balance of the tumor microenvironment may be the key to its tumor‐promoting effect under the influence of IGFL2‐AS1 in CRC.

As it is well established, the promoter of *CA9* is transactivated under various stimuli that activate HIF‐1α.^21^ Activation of the CA9 promoter is hardly seen in the absence of HIF‐1α because the transcriptional activity of CA9 depends on the tight regulation of the hypoxia‐responsive element containing the HIF‐binding site close to the transcription start site; therefore, HIF‐1α is the most pivotal transcription factor of CA9.^18^ Interestingly, we observed that the expression pattern of IGFL2‐AS1 and HIF‐1α was consistent in CRC cells. By knocking down HIF‐1α in cells overexpressing IGFL2‐AS1, we found that CA9 expression was decreased compared with that in the treatment group overexpressing IGFL2‐AS1 alone. Moreover, IGFL2‐AS1 could upregulate the HIF‐1α expression level by inhibiting its ubiquitinated proteasomal degradation. This validates the hypothesis that the regulation of CA9 by IGFL2‐AS1 is mediated by HIF‐1α. Nonetheless, there are some limitations in this study. On the one hand, all experimental data were obtained under normoxic conditions, so it is unclear whether the effect of IGFL2‐AS1 on the HIF‐1α/CA9 pathway also exists under hypoxic conditions. On the other hand, it is not clear whether how IGFL2‐AS1 affects HIF‐1α degradation is direct or indirect.

HIF is a heterodimeric complex composed of the O_2_‐reactive functional subunit α and the stable structural subunit β, and its activity is mainly controlled by the stabilization of the α subunit (HIF‐1α, HIF‐2α, or HIF‐3α).^34^ When oxygen is abundant, the HIF‐1α protein is translated but rapidly degraded. The main mechanism involves, as mentioned above, the degradation of HIF‐1α modified by specific prolyl hydroxylase (PHD) by VHL via the ubiquitin‐proteasome system.^35^ Several mechanisms have also been proposed to explain the degradation pathway of HIF‐1α. For example, the receptor of activated protein kinase C (RACK1) can compete with heat shock protein 90 (HSP90) for binding to the PAS‐A domain of HIF‐1α, thereby accelerating the O_2_/PHD/VHL independent proteolysis of HIF‐1α.^36^ Activation of p53 has also been shown to accelerate proteasome‐dependent HIF‐1α degradation and downregulate CA9.^37^ We have noted that the proteolytic mechanism of HIF‐1α is complex, and the specific mode of action of IGFL2‐AS1 on HIF‐1α could be further researched. Furthermore, due to elevated oncogenic signaling in tumor cells, HIF‐1α expression can also be regulated in an oxygen‐independent manner, including effects on its mRNA transcription and translation.^34^ This can result in high HIF‐1α expression levels even under normoxic conditions. According to previous reports, treatment with insulin‐like growth factor 1 (IGF1) can induce the protein synthesis of HIF‐1α in cells.^38^ The IGFL family has a similar protein structure and expression pattern to the IGF family,^39^ and previous reports have confirmed that IGFL2‐AS1 could promote IGFL1 expression in tumor cells.^11^ Therefore, although our data suggest that IGFL2‐AS1 may primarily affect the post‐transcriptional regulatory pathway of HIF‐1α, whether it also acts on the protein synthesis of HIF‐1α through IGFL1 in CRC needs further investigation.

**FIGURE 8:**
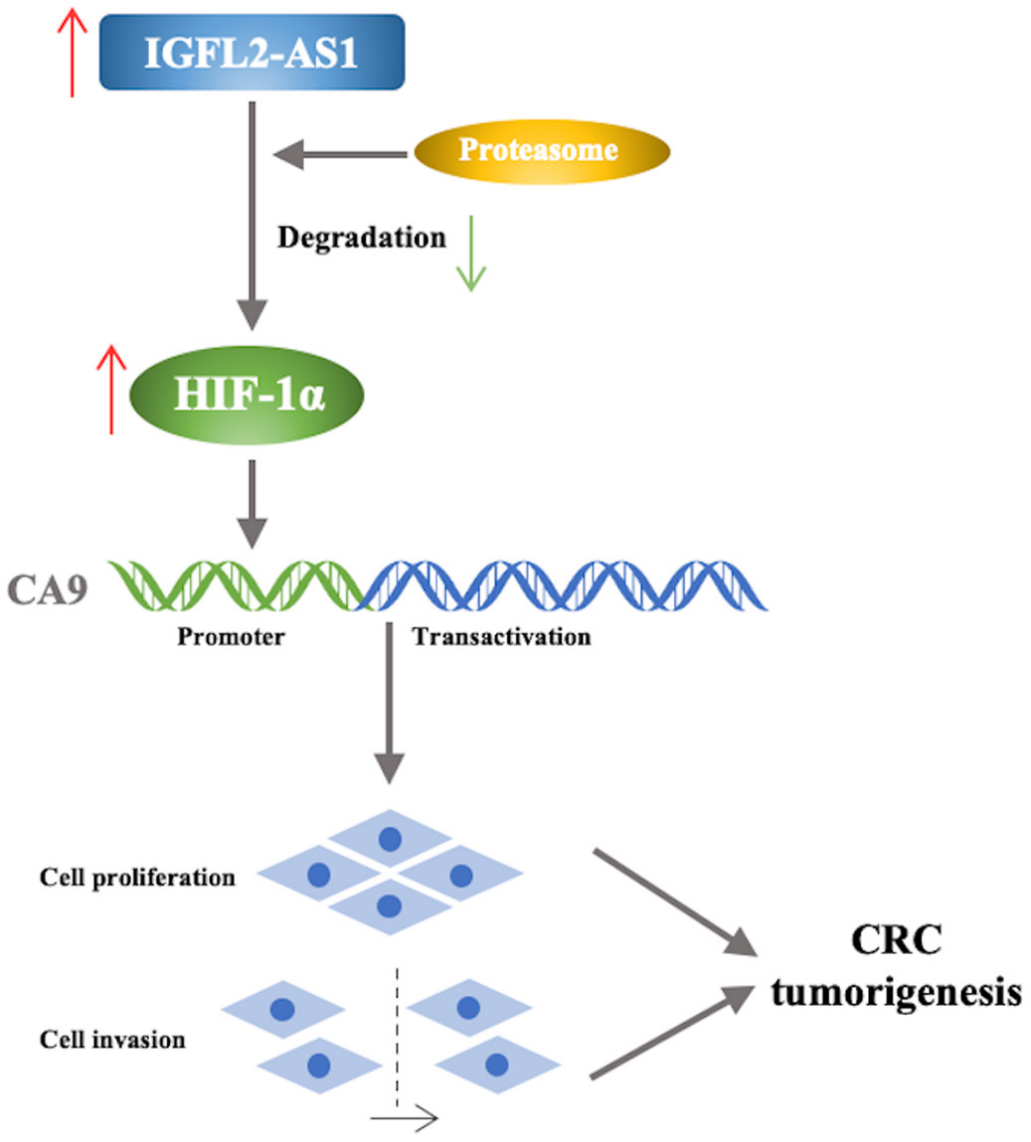
The schematic illustration of a model for IGFL2‐AS1 to promote tumor growth in CRC. IGFL2‐AS1 is upregulated in CRC and may inhibit the proteasomal degradation of HIF‐1α, which could increase HIF‐1α expression and its downstream gene CA9, thereby promoting CRC cell proliferation, migration, and invasion

In conclusion, we demonstrated that the lncRNA IGFL2‐AS1 often has a tendency toward overexpression in CRC, and its expression level is an important factor affecting CRC cell proliferation, migration, and invasion. Inhibition of HIF‐1α degradation by IGFL2‐AS1 results in the upregulation of CA9, which then leads to the promotion of CRC growth both in vivo and in vitro (Figure 8). Consequently, as an oncogenic factor in CRC, IGFL2‐AS1 is expected to become a new valuable therapeutic target and prognostic indicator for CRC.

## AUTHOR CONTRIBUTIONS

**Mengdi Qin:** Conceptualization (equal); data curation (lead); formal analysis (lead); software (lead); visualization (lead); writing – original draft (lead); writing – review and editing (equal). **Qiang Liu:** Data curation (supporting); formal analysis (supporting); investigation (equal). **Wei Yang:** Data curation (equal); resources (equal). **Qiaofeng Wang:** Resources (equal). **Zheng Xiang:** Conceptualization (equal); funding acquisition (lead); project administration (lead); resources (equal); writing – review and editing (lead).

## CONFLICT OF INTEREST

The authors declare that they have no competing interests.

## ETHICS STATEMENT

This study was conducted in accordance with the principles embodied in the Declaration of Helsinki. The experimental protocol involving human tissues for this study was approved by the Ethics Committee of the First Affiliated Hospital of Chongqing Medical University. Written informed consent was obtained from each human tissue provider. The animal experiment protocol of this study was authorized by the Animal Experiment Ethics Committee of Chongqing Medical University.

## Supporting information


Table S1.
Table S2.
Table S3.
Figure S1.
Figure S2.
Figure S3.

## Data Availability

The sequence read data in this study were submitted to the NCBI Sequence Read Archive (SRA) database (accession: SRR20011881, SRR20011880). The rest of the datasets generated and analyzed during this study are included in the article and Supplementary material. Further inquiries can be directed to the corresponding author.
