# Supplementary material for: IGFL2‐AS1‐induced suppression of HIF‐1α degradation promotes cell proliferation and invasion in colorectal cancer by upregulating CA9
Source: Cancer Med. 2022 Dec 20;12(7):8415–32. doi: 10.1002/cam4.5562 (PMC10134350; doi:10.1002/cam4.5562)

***Supplementary Materials***

**1.Supplementary Table**

**Table S1. primer sequences of qRT-PCR**

| Primer | Sequences (5'-3') |
| --- | --- |
| GAPDH F | AGAAGGCTGGGGCTCATTTG |
| GAPDH R | AGGGGCCATCCACAGTCTTC |
| IGFL2-AS1 F | TGACCTATGGACTCAGGAGC |
| IGFL1-AS1 R | TTGGGGCTGAAATCAGGAACT |
| U6 F | CTCGCTTCGGCAGCACA |
| U6 R | AACGCTTCACGAATTTGCGT |
| SEMA5B F | TGCGGCGTGGAGTTCAAGA |
| SEMA5B R | GTCCTCGTCTCGGTCCTTCT |
| SLITRK6 F | AAGCAAGAACACAGTGACAGATGACA |
| SLITRK6 R | TGGCACAGCGTCCGTAAGAGAT |
| PCK1 F | GGATGACATTGCCTGGATGAA |
| PCK1 R | GTGTTCTTCTGGATGGTCTTGA |
| NDUFA4L2 F | GCTTTACTTGCTGCGACTC |
| NDUFA4L2 R | AGGAACTTGTATTGGTCATTGG |
| CA9 F | CCTGGCTGCTGGTGACATCCTA |
| CA9 R | CCTTCTGTGCTGCCTTCTCATCTG |
| BDKRB2 F | GCAGAAGTTCAAGGAGATCCAGACAGAG |
| BDKRB2 R | GAATAGCAGCAGCACAACCAGGACTA |
| PPFIA4 F | ATGGAGCGGCAGAAGAGGAG |
| PPFIA4 R | CGAGTTCAGTCACGGTTGTTGT |
| SVEP1 F | GTCTCCTGCTACACATTCAT |
| SVEP1 R | GCCATTCCAAGTTCCATCT |
| EGLN3 F | ATGCCCCTGGGACACATC |
| EGLN3 R | GTCTTCAGTGAGGGCAGA |

**Table S2.shRNA target sequences**

| shRNA | sequence( 5'-3') |
| --- | --- |
| shIGFL2-AS1#0 | GAAGAAGTGGCCAGGAAATTA |
| shIGFL2-AS1#1 | GGCACAAGCTGTTCCAGTATA |
| shIGFL2-AS1#2 | GAGTGTGAGCCTTCTATTATG |
| shHIF-1α | GCCAGATCTCGGCGAAGTAAA |
| sh-nc | ﻿TTCTCCGAACGTGTCACGT |

**Table S3.** **Top 50 upregulated lncRNAs in CRC (ordered by logFC)**

| lncRNA | logFC | logCPM | PValue | FDR |
| --- | --- | --- | --- | --- |
| LINC02418 | 8.9707229 | 4.17904086 | 1.54E-47 | 4.37E-46 |
| FEZF1-AS1 | 8.90005691 | 2.29056134 | 1.29E-42 | 2.97E-41 |
| AC026336.3 | 8.43714729 | 0.92927823 | 3.80E-24 | 3.33E-23 |
| BX322234.2 | 8.23224647 | -0.0053656 | 2.72E-10 | 8.68E-10 |
| AC105460.1 | 8.11472978 | 1.77811068 | 1.02E-11 | 3.68E-11 |
| AC104823.1 | 7.86171141 | 2.1896827 | 5.21E-24 | 4.52E-23 |
| TMEM132D-AS1 | 7.81216819 | 0.07142568 | 1.06E-10 | 3.50E-10 |
| FP671120.3 | 7.77895871 | 0.81508906 | 5.69E-08 | 1.47E-07 |
| AL355075.4 | 7.65770886 | 1.51813389 | 1.25E-09 | 3.78E-09 |
| LINC01234 | 7.6133288 | 2.07341915 | 7.58E-27 | 7.85E-26 |
| LINC02474 | 7.52465607 | -0.0377817 | 2.73E-18 | 1.63E-17 |
| AC073365.1 | 7.49913041 | -0.5051732 | 7.63E-20 | 5.00E-19 |
| AC055717.2 | 7.44778569 | -0.2631473 | 4.69E-35 | 7.50E-34 |
| AC079466.1 | 7.36286595 | 0.15761372 | 1.40E-15 | 6.93E-15 |
| AC007099.1 | 7.33852371 | -0.1280511 | 1.51E-41 | 3.30E-40 |
| LINC02163 | 7.23563476 | -0.4678262 | 7.81E-57 | 3.26E-55 |
| RMRP | 7.00548946 | -0.4337442 | 1.73E-08 | 4.69E-08 |
| AC007608.2 | 6.92727207 | -0.730485 | 7.40E-13 | 2.93E-12 |
| LINC00460 | 6.87126213 | 1.00801625 | 6.27E-38 | 1.16E-36 |
| LINC01705 | 6.817321 | -0.5053507 | 1.69E-36 | 2.91E-35 |
| LINC01411 | 6.79243146 | 1.02705485 | 6.22E-23 | 5.02E-22 |
| NPSR1-AS1 | 6.68500338 | 0.2210181 | 1.90E-27 | 2.02E-26 |
| LINC02582 | 6.6615788 | -0.6222966 | 8.95E-10 | 2.73E-09 |
| ERVMER61-1 | 6.63447385 | -0.9615058 | 6.44E-12 | 2.35E-11 |
| LINC02577 | 6.62571462 | 0.88383492 | 5.32E-54 | 1.94E-52 |
| AL162413.1 | 6.62183384 | -1.1802448 | 3.24E-21 | 2.33E-20 |
| LINC01602 | 6.34761214 | -1.1507879 | 7.86E-20 | 5.15E-19 |
| LINC01811 | 6.34626269 | -0.303511 | 7.32E-32 | 9.92E-31 |
| AC097478.1 | 6.32195406 | 1.2411589 | 1.02E-15 | 5.11E-15 |
| LINC01419 | 6.30160558 | -1.7596939 | 1.78E-10 | 5.79E-10 |
| LINC02042 | 6.12907976 | -1.5023924 | 1.92E-09 | 5.69E-09 |
| LINC00659 | 6.1214532 | 0.60291662 | 6.70E-45 | 1.71E-43 |
| AC003958.2 | 6.07687104 | -1.9508632 | 5.19E-16 | 2.66E-15 |
| AC005256.1 | 6.06947121 | -1.1976449 | 1.19E-26 | 1.22E-25 |
| LINC00858 | 6.05114524 | 0.73131104 | 1.05E-36 | 1.83E-35 |
| AC123023.1 | 6.04471102 | 0.0332731 | 3.81E-40 | 7.86E-39 |
| AL162582.1 | 6.02745034 | -0.1557123 | 1.10E-24 | 9.94E-24 |
| AFAP1-AS1 | 6.02225686 | 3.75288858 | 8.78E-16 | 4.42E-15 |
| AL161431.1 | 5.99794126 | 3.25941131 | 1.21E-29 | 1.46E-28 |
| IGFL2-AS1 | 5.96899652 | 0.21567834 | 1.24E-19 | 8.00E-19 |
| AC117386.2 | 5.84306642 | -1.2989148 | 1.78E-25 | 1.69E-24 |
| PCAT14 | 5.84146655 | 1.24155308 | 1.64E-12 | 6.35E-12 |
| AC135388.1 | 5.83685336 | -2.1537437 | 9.57E-21 | 6.64E-20 |
| LINC02257 | 5.77649425 | -1.5456015 | 2.69E-26 | 2.70E-25 |
| PLAC4 | 5.7498909 | 2.52644795 | 1.71E-21 | 1.25E-20 |
| FIRRE | 5.72465206 | 0.86341845 | 2.05E-34 | 3.15E-33 |
| AC025154.2 | 5.69332262 | -0.6250276 | 4.23E-28 | 4.68E-27 |
| AL445223.1 | 5.67695608 | -2.0218945 | 6.34E-09 | 1.79E-08 |
| AC012501.1 | 5.66479711 | -2.0939323 | 6.33E-17 | 3.45E-16 |
| LINC02253 | 5.64503508 | 1.28879827 | 1.56E-30 | 1.97E-29 |

**2. Supplementary Figure**

**Fig. S1** The apoptosis ability of LoVo cells was measured by flow cytometry.

**
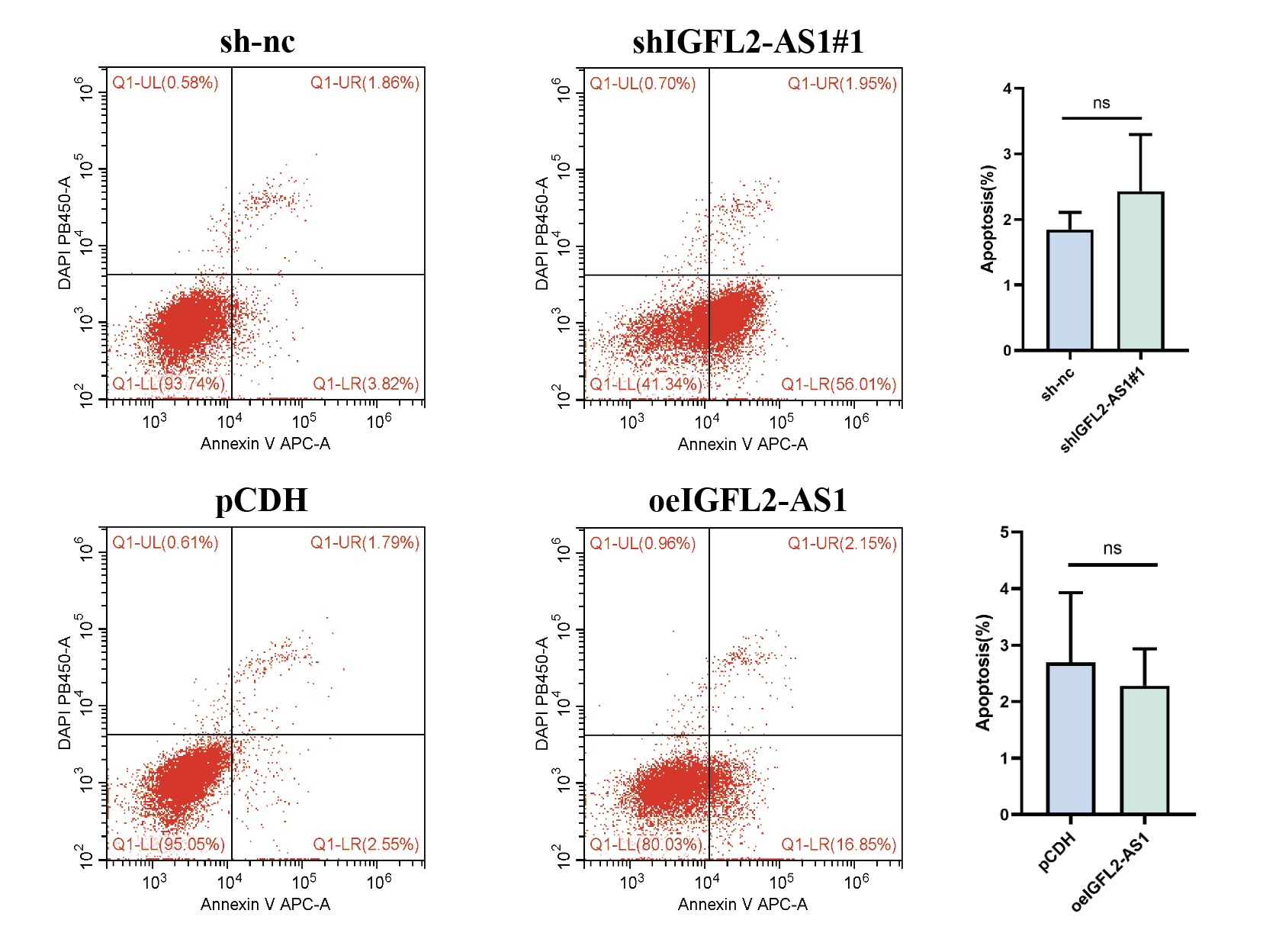
**

**Fig. S2** qRT-PCR was used to measure CA9 mRNA level in CRC cells after IGFL2-AS1 knockdown with or without CA9 overexpression


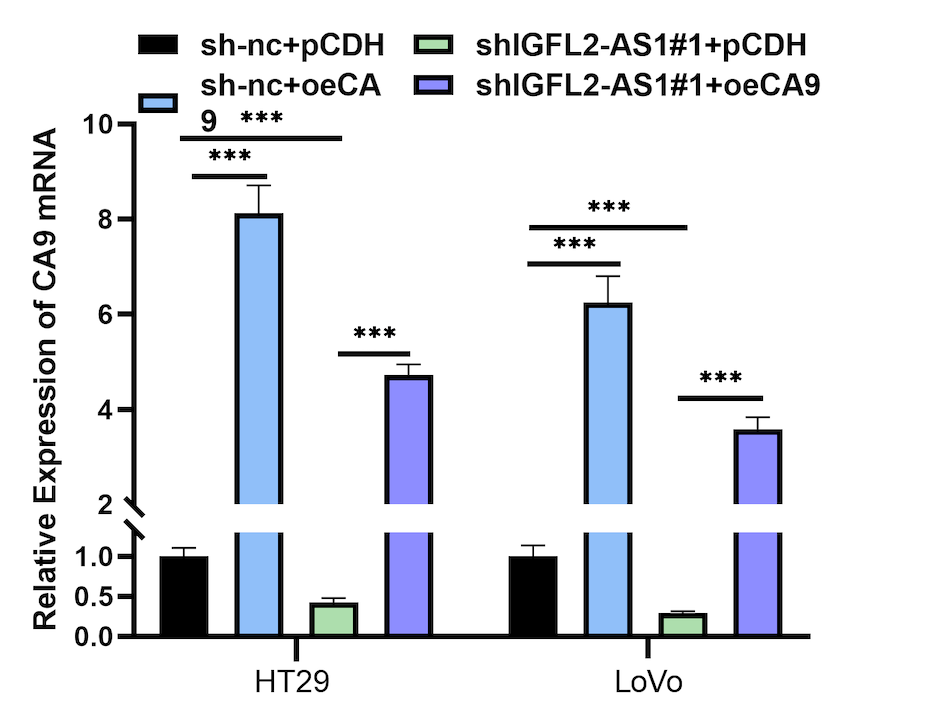


**Fig. S3** The result of qRT-PCR showed that IGFL2-AS1 had no significant effect on the mRNA level of HIF-1α


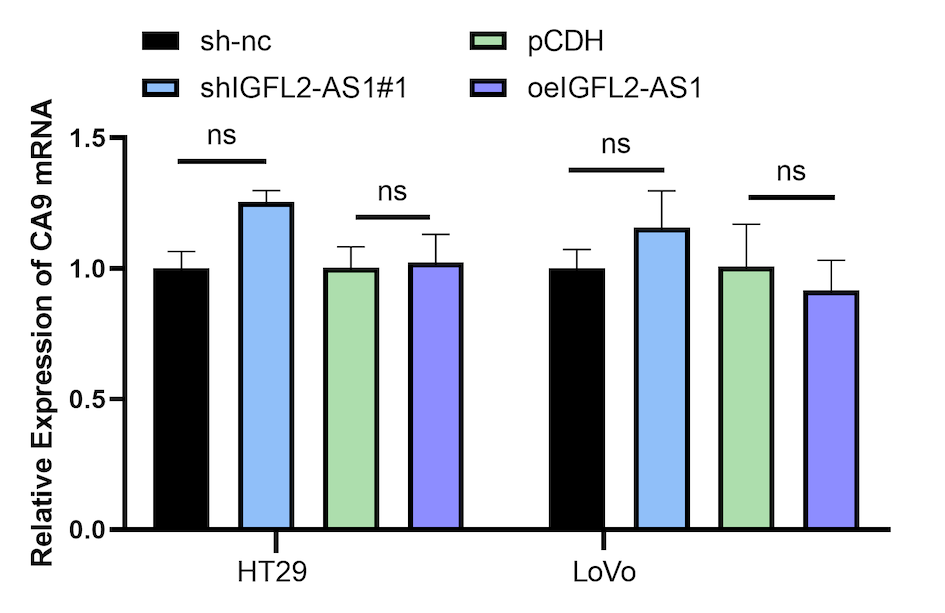

Supplement: Supplementary file 1 — Table S1. Table S2. Table S3. Figure S1. Figure S2. Figure S3. [file CAM4-12-8415-s001.docx]
